# Chestnut-Derived Activated Carbon as a Prospective Material for Energy Storage

**DOI:** 10.3390/ma13204658

**Published:** 2020-10-19

**Authors:** Katarzyna Januszewicz, Anita Cymann-Sachajdak, Paweł Kazimierski, Marek Klein, Justyna Łuczak, Monika Wilamowska-Zawłocka

**Affiliations:** 1 Department of Energy Conversion and Storage, Faculty of Chemistry, Gdańsk University of Technology, Narutowicza 11/12, 80-233 Gdańsk, Poland; katjanus@pg.edu.pl (K.J.); anita.cymann@pg.edu.pl (A.C.-S.); 2 Institute of Fluid Flow Machinery, Polish Academy of Sciences, 80-233 Gdańsk, Poland; pkazimierski@imp.gda.pl (P.K.); marek.klein@imp.gda.pl (M.K.); 3 Department of Process Engineering and Chemical Technology, Faculty of Chemistry, Gdańsk University of Technology, Narutowicza 11/12, 80-233 Gdańsk, Poland; justyna.luczak@pg.edu.pl

**Keywords:** biomass-derived activated carbons, pyrolysis, activation process, energy storage, electrochemical capacitors

## Abstract

In this work, we present the preparation and characterization of biomass-derived activated carbon (AC) in view of its application as electrode material for electrochemical capacitors. Porous carbons are prepared by pyrolysis of chestnut seeds and subsequent activation of the obtained biochar. We investigate here two activation methods, namely, physical by CO_2_ and chemical using KOH. Morphology, structure and specific surface area (SSA) of synthesized activated carbons are investigated by Brunauer-Emmett-Teller (BET) technique and scanning electron microscopy (SEM). Electrochemical studies show a clear dependence between the activation method (influencing porosity and SSA of AC) and electric capacitance values as well as rate capability of investigated electrodes. It is shown that well-developed porosity and high surface area, achieved by the chemical activation process, result in outstanding electrochemical performance of the chestnut-derived porous carbons.

## 1. Introduction

In recent years, biomass-derived carbon materials have been widely studied due to their structural diversity and attractive properties, which may be tailored by various preparation procedures and activation methods [1,2,3,4,5,6]. Porous carbonaceous materials including activated carbons (AC) due to their chemical stability, superior electrical conductivity, high specific surface area, large pore volume and specific pore structure are used in many application: photochemical degradation [7,8], catalyst carrier, adsorption [9,10], carrier of a phase change materials (PCMs) for application in building materials [11], fuel cells [12], energy conversion and storage [13,14,15]. A popular research direction is to reuse biomass as an environmentally friendly, easily accessible, low-cost and renewable carbon precursor.

Biomass can be transformed into valuable carbon materials by thermochemical conversion processes performed in various oxygen conditions, e.g., gasification, pyrolysis and combustion [12]. All of these methods involve numerous chemical reactions, such as decomposition, dehydration, oxidation, polymerization etc. Taking into account the variability of the raw materials composition and thermal transformation complexity, the mechanism of these processes is often difficult to thoroughly investigate. During pyrolysis, the biomass is heated in a non-reactive atmosphere. Decomposition of the lignocellulosic materials starts at 350 °C and goes up to 700–800 °C in the absence of air/oxygen. The polymeric compounds forming biomass undergo transformation into smaller molecules forming gases, condensable vapours (tars and oils) and solid biochar [16,17,18,19].

The surface of the solid product is rich in various functional groups: alkyl, carbonyl, carboxyl etc. Nevertheless, its surface area is relatively low and pore structure not expanded enough for electrochemical applications. In this regard, the activation processes are used to unblock and develop micropores, thus expanding the surface area and modifying the chemical properties of the surface. Both known methods, namely chemical and physical activation, are based on penetration/treatment of carbon material structure by activation agents such as oxygen plasma, KOH, NaOH, HNO_3_, K_2_CO_3_, steam, CO_2_, ZnCl_2_ at high temperature (up to 800 °C).

Activated carbons of developed porosity are valuable electrode materials for supercapacitor application [20,21,22]. Biomass as a precursor for electrode material has to fulfil several criteria, in particular, biochar should have: (i) high carbon content (to provide good electric conductivity), (ii) low amount of impurities (to avoid further purification and limit side reactions during operation of the device), (iii) high surface area (to assure high electric double-layer capacitance) and (iv) tailored porosity (with pore sizes accessible for ions). To meet those criteria, the obtained biochar has to be activated in order to remove impurities and pyrolysis residues as well as increase its specific surface area (SSA) through the creation of micro- and mesopores. Biomass type, pyrolysis conditions and further activation processes have a significant influence on the final properties of activated carbons. Therefore, it is crucial to investigate a whole range of different biomass wastes, optimize the thermal treatment parameters, select appropriate activation method and optimize its conditions [5]. Recently, a number of species, such as agricultural and forestry biomass [23,24], soybean pod [25], castor shell [26], waste biomass [27], durian husk [28], cassava stalks and bamboo [29], rice straw [30], chestnut shell [12,31,32,33,34,35,36] and water chestnut (*Eleocharis dulcis*) [37,38] have been investigated as porous carbon precursors for energy storage application.

Focusing on activated carbon from the chestnut plant family and different parts of chestnut fruit, several works refer to their application as electrodes for supercapacitors. Jiang et al. studied KOH-activated carbon obtained from chestnut shells [32]. They investigated five samples prepared with various ratios between pre-carbonized material and KOH. The highest SSA of 1829.7 m^2^ g^−1^ was achieved for the sample activated with a 1:3 biochar: KOH ratio, which resulted in the highest capacitance of 238.2 F g^−1^. Cheng et al. prepared activated carbons by carbonization of ZnCl_2_ pre-treated chestnut shells [33]. The resulted AC showed SSA up to 1987 m^2^ g^−1^, which resulted in the capacitance value of 105.4 F g^−1^. Potassium sulphate as the activating agent was used by Hong et al. for preparation of chestnut shell-derived AC [34]. This led to AC with a specific surface area of 1412 m^2^ g^−1^ and capacitance value equal to 265 F g^−1^ at a relatively low current density of 0.1 A g^−1^. Another activating agent—melamine—was used by Wan et al. for activation of chestnut shell-derived carbons [35]. They achieved a capacitance value of 402.8 F g^−1^ at 0.5 A g^−1^ for the sample with SSA of 691.8 m^2^ g^−1^. The high-performance supercapacitor electrode with the KHCO_3_ activated carbon from the chestnut shell was tested by Hong et al. [31]. The activation of biochar with potassium bicarbonate resulted in high surface area porous carbons (2298 m^2^ g^−1^), which exhibit capacitance of 387 F g^−1^ (at 2 A g^−1^) and exceptional cycle stability of 98.68% after 10,000 charge-discharge cycles at 30 A g^−1^ [31]. Nitrogen-doped porous carbon was obtained from edible Chinese water chestnut corms by Wei et al. [37]. The raw biochar was chemically activated with KOH at 600–900 °C for 2 h, which resulted in the N-doped AC characterized by a large surface area of 3401 m^2^ g^−1^. This, in turn, led to the high specific capacitance (346 F g^−1^), energy density (22.4 Wh kg^−1^ at 0.5 A g^−1^) and good cycling stability (capacitance retention of 97.6% after 5000 cycles at 1 A g^−1^) [37].

Activated carbons derived from various types of chestnuts were also investigated for several other applications [9,10,39,40,41,42,43]. For instance, chestnut shells served as a raw material for the preparation of biochar with high calorific value as a potential fuel [39]. Chestnut cupulae was used by Kar et al. for the preparation of bio-oil, serving as fuel or chemical feedstock [40]. Dyjakon et al. studied forest biomass containing horse chestnuts as an alternative fuel [41]. Biochar from chestnut shells supported with carbon nanotubes was examined as an adsorbent for heavy metals contaminants by Yang et al. [9]. The adsorption capacity of malachite green oxalate was tested by Tzvetkov et al. using horse chestnut-derived biochar activated by mechanochemical and chemical processes [10].

In this work, we pyrolyze and activate, by two different methods (physical with CO_2_ and chemical using KOH), the horse chestnut seeds (*Aesculus hippocastanum* L.). To the best of our knowledge, activated carbons, obtained from this type of biomass, have not been investigated as electrode materials for supercapacitors. Here we show the influence of activation method on the morphology, structure, surface area and the resulted capacitive properties of the synthesized porous carbons. A thorough electrochemical study of the chestnut-derived AC reveals the potential of this material for application in high-power energy storage devices.

## 2. Materials and Methods

### 2.1. Materials

In this work, waste chestnuts were used to obtain activated carbon as a potential material for energy storage. This type of biomass was selected because a horse chestnut (*Aesculus hippocastanum* L.)*,* with inedible, large seeds (chestnuts), is a popular tree in the Gdańsk urban area.

Hydrochloric acid and potassium hydroxide (Avantor Performance Materials Poland S.A., Gliwice, Poland) were of analytical grade and used as received. Electrolyte for electrochemical studies (6 M KOH) was prepared using deionized water. Polytetrafluoroethylene (PTFE) dispersion in water (60 wt%) was obtained from Sigma Aldrich (Merck KGaA, Darmstadt, Germany).

### 2.2. Pyrolysis and Activation Process of Biochar

**Figure 1:**
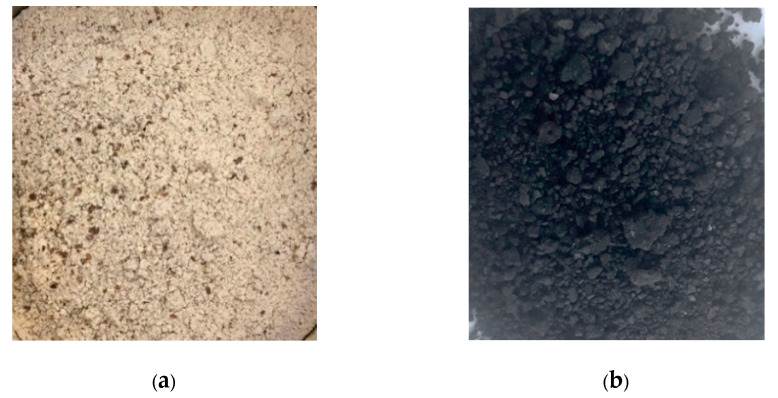
(**a**) Ground chestnut before pyrolysis, (**b**) biochar—chestnut after pyrolysis process.

The methodology of preparation of biochar and activated carbon was described in details in our previous work [11]. Briefly, before the pyrolysis process, the chestnuts were dried, ground using a knife mill in two stages (without a sieve and using a sieve with a mesh diameter of 3 mm). The raw material is shown in Figure 1a. The pyrolysis process was conducted in a batch reactor located inside a laboratory-scale furnace (Neoterm MidiSUN lift 3.0 with thermoregulatory KXP3, Wrocław, Poland). The biochar sample (Figure 1b) was obtained by fast pyrolysis process (heating rate 100 °C min^−1^, dwell time 30 min. at 800 °C) under limited access of air without inert gas flow. The obtained biochar sample was activated using two different methods: chemical (KOH) and physical (CO_2_).

For chemical activation, the biochar sample was mixed with solid KOH with a mass ratio of 1:3 and ground by mortar. The amount of an activating agent was selected based on literature reports [44,45] where the dependence of the KOH amount on the surface area of activated carbon was described. The activation process took place in a quartz tube inside the horizontal ceramic furnace, under controlled nitrogen flow (flow rate of 50 mL min^−1^) and temperature (800 °C for 1 h, the heating rate of 10 °C min^−1^). The biochar after activation process (5 g) was put into a beaker with distilled water (200 mL), sonicated for 30 min, and left for sedimentation for 24 h. The suspension was filtered under reduced pressure, and the filtrate was washed with water and 5 M HCl alternately until a neutral pH of the filtrate was achieved. The activated biochar was dried at 105 °C overnight.

The physical activation process of chestnut char was conducted in the steal horizontal reactor placed in a ceramic furnace (ALGA, Gdańsk, Poland) equipped with resistance heaters, thermal insulation and a control system. The temperature of the process was equal to 800 °C (heating rate of 10 °C min^−1^, dwell time 1 h), and the CO_2_ flow was constant (10 dm^3^ h^−1^) for the whole process. The amount of activation agent was set as the 0.5:1 molar ratio between the activated agent CO_2_ and C content in biochar (C_biochar_-based on the elemental analysis of the biochar sample (Table 1)). A 1:1 molar ratio was also checked, but the higher amount of activated agent caused too intensive oxidation of the chestnut-derived biochar and resulted in a lower SSA (21.2 compared to 105.7 m^2^ g^−1^ for 1:1 and 0.5:1 CO_2_:C_biochar_ ratio, respectively). However, it is worth noticing that the amount of activated agent needed for the effective activation depends largely on the type of biomass as well as the structure and chemical composition of biochar [46].

### 2.3. Characterization Techniques

Elemental composition of the raw and pyrolyzed materials was determined using the CHNS-O analyzer Flash 2000 (Thermo Scientific, Waltham, MA, USA). Proximate analysis of the samples, including determination of ash (PN-EN 15,403:2011) and volatile matter (PN-EN 15,402:2011), was performed in the muffle furnace (LIFT3.0 + KXP4 R, Neoterm, Wrocław, Polska). Moisture content was measured by a moisture analyzer (Mettler Toledo, Greifensee, Switzerland). The morphology of the activated biochars was studied by scanning electron microscopy (SEM) using a Phenom^TM^ XL G2 Desktop SEM (Thermo Fischer Scientific, Waltham, MA, USA) with the accelerating voltage of 10 kV. The microtextural characteristic of the activated samples was determined using the Brunauer-Emmett-Teller (BET) method by N_2_ adsorption–desorption isotherms at 77 K using a Micromeritics Gemini V200 Shimadzu (Kyoto, Japan) analyzer.

Electrochemical measurements were carried out using potentiostat/galvanostat SP-200 (BioLogic, Grenoble, France). The materials were electrochemically characterized by three electrochemical techniques: cyclic voltammetry at scan rates 5–500 mV s^−1^, galvanostatic charge-discharge (GCD) at current densities 0.025–10 A g^−1^ and electrochemical impedance spectroscopy at frequency range 100 kHz–10 mHz with the amplitude V_RMS_ equal to 10 mV. For long-term cycling, GCD at the current density of 1 A g^−1^ was used. All the electrochemical investigations were carried out in the symmetric two-electrode configuration in SWAGELOK^®^ type cells made of polytetrafluoroethylene (PTFE) with the stainless steel current collectors. The glass fibre with a thickness of 300 µm (Marcheley–Nagel, MN GF-1), soaked with 6 M KOH electrolyte served as a separator.

### 2.4. Electrode Preparation

The electrodes for measurements were in the form of pellets prepared as follows: 80 wt.% of chestnut-derived activated carbon, 10 wt.% of PTFE binder and 10 wt.% of carbon black (used as a conductive additive) were mixed with isopropyl alcohol and stirred at elevated temperature (120 °C). After evaporation of the excess solvent, the obtained dough was rolled to form the electrode sheet (about 200 µm in thickness) and dried at 60 °C. Then, the electrodes with a diameter of 6 mm were cut as self-standing disks. The mass loading of the electrodes ranged from 7 to 14 mg cm^−2^. Electrodes with similar masses were chosen to assemble the symmetric cells.

## 3. Results and Discussion

### 3.1. CHNS and Proximate Analyses

The proximate analysis of chestnut as a raw material was presented in our previous work [11]. The ash and volatile content was 3.5 wt.% and 74.6 wt.%, respectively. From 128.1 g of raw chestnut biomass, 30.5 g of solid fraction remained after the pyrolysis process. Low efficiency of the carbonization process (23.8%) is due to a high amount of starch (hydrocarbon) in this type of biomass, which degrades at elevated temperature, mostly to volatile compounds (74.6 wt.%). The chemical composition of the raw material, the resulted biochar and activated carbons (Table 1) proved that the investigated chestnut type did not contain any inorganic impurities, such as chlorine or metals. The carbon content increased after pyrolysis from 45.97 wt.% to 75.14 wt.%, whereas the amount of other elements such as oxygen, hydrogen and sulfur decreased. Carbon content in the activated samples was equal to 75.04 and 60.54, for CO_2_ activated and KOH activated sample, respectively. Carbon and oxygen contents for the sample activated with CO_2_ were very similar to the starting biochar sample, whereas K–OH activated sample differs significantly. Lower carbon content in C–KOH sample compared to C-non may result from a higher degree of oxidation but may also come from impurities remaining after the activation process.

### 3.2. Surface Area Analysis

The effectiveness of both activation processes (chemical and physical) was assessed by comparing the results of the SSA and total pore volume of the samples [11]. The analysis was performed according to the BET theory for all the samples and presented in Table 2. The chestnut raw material sample, before the activation process, had an SSA of 17.1 m^2^ g^−1^ and a total pore volume of 0.0094 cm^3^ g^−1^. After the physical activation of this sample, a significant increase of SSA from 17.1 m^2^ g^−1^ to 105.7 m^2^ g^−1^ was observed. On the other hand, the SSA and the total pore volume, after KOH activation, were equal to 1221.2 m^2^ g^−1^ and 0.625 cm^3^ g^−1^, respectively. The results reveal much higher efficiency of chemical activation compared to the physical one. This can be explained by the mechanism of carbon degradation that occurs during the chemical activation process. KOH activation is a complex process with many variables depending on precursor type, its reactivity and experimental conditions. Therefore, the mechanisms of KOH activation, proposed by different groups of researchers, slightly differ from each other [47,48,49]. However, it is established that the main stages, occurring below 700 °C, are as follows: dehydration (2 KOH = K_2_O + H_2_O); water-gas reaction (C + H_2_O = H_2_ + CO); water-gas shift reaction (CO + H_2_O = H_2_ + CO_2_); and carbonate formation (K_2_O + CO_2_ = K_2_CO_3_) [48,49]. At temperatures above 700 °C, a formation of metallic potassium is observed due to K_2_O reduction with hydrogen or carbon (K_2_O + H_2_ = 2K + H_2_O; K_2_O + C = 2K + CO) [49]. Metallic K causes a deeper penetration of the activation agent in the carbon material. Moreover, the SSA is also influenced by the presence of functional groups formed during the chemical activation of carbon.

### 3.3. SEM Analysis

**Figure 2:**
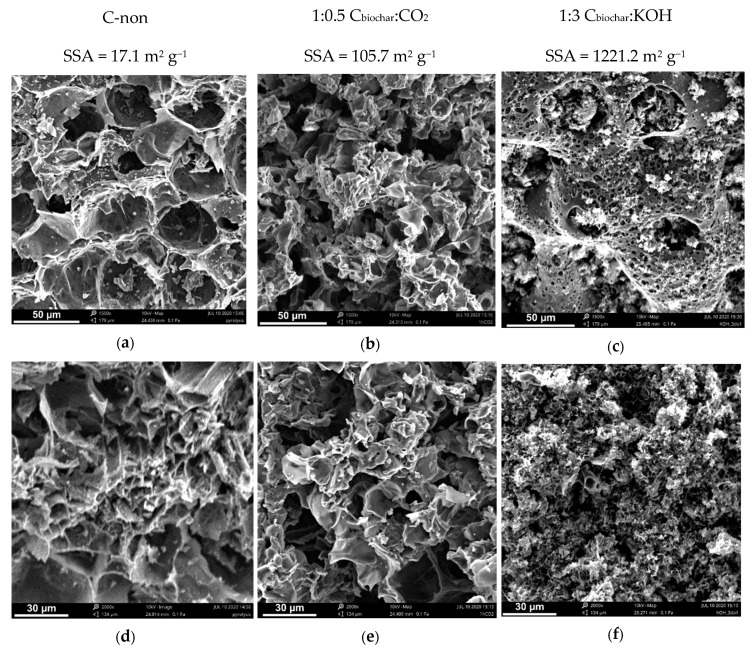
SEM images of: raw biochar (**a**,**d**); biochar after physical activation (**b**,**e**); biochar after chemical activation (**c**,**f**).

Scanning electron microscopy was used to investigate the morphology of the biochar before and after the activation process. SEM images with the corresponding specific surface area values of the samples are shown in Figure 2. The biochar sample is characterized by a small number of large pores, which results in low SSA (17.1 m^2^ g^−1^). Besides, surface contamination of raw biochar is noticed in the form of small particles on the surface being residues (e.g., ash, organic impurities such as aromatic compounds, hydrocarbons and polycyclic aromatic hydrocarbons (PAHs)) after the pyrolysis process. During the activation process, the structure of the samples and their SSA change noticeably. The physical activation with CO_2_ increases specific surface area to the value of 105.7 m^2^ g^−1^, which changes the structure of the sample; smaller pores appear, and the number of surface contamination particles decreases (Figure 2b,e). The chemical activation of biochar has a much more significant impact on the structure and surface area than the physical activation. Substantially more pores of smaller diameter and various depth can be observed in SEM images of KOH activated carbon (Figure 2 c,f), which results in two orders of magnitude higher SSA (1221.2 m^2^ g^−1^) compared to the pristine biochar sample. Moreover, the edges of AC particles are smoother, and the structure resembles a sponge, which is the result of the deeper penetration of the strong activating agent.

### 3.4. Electrochemical Analysis Results

Electrochemical performance of chestnut-based activated carbons was conducted in a two-electrode system with 6 M KOH electrolyte. The specific capacitance (*C_s_*) values were calculated from cyclic voltammograms and galvanostatic charge/discharge according to the Formulas (1) and (2):

(1) $$C_{s}=2\cdot \frac{i}{m\cdot \nu },$$

where: *i*—current recorded in cyclic voltammetry; *m*—active mass of one electrode; and ν—scan rate.

(2) $$C_{s}=2\cdot \frac{i\cdot \Delta t}{m\cdot U},$$

where: *i*—discharge current; *m*—active mass of one electrode; Δ*t*—discharge time; and *U*—cell voltage.

**Figure 3:**
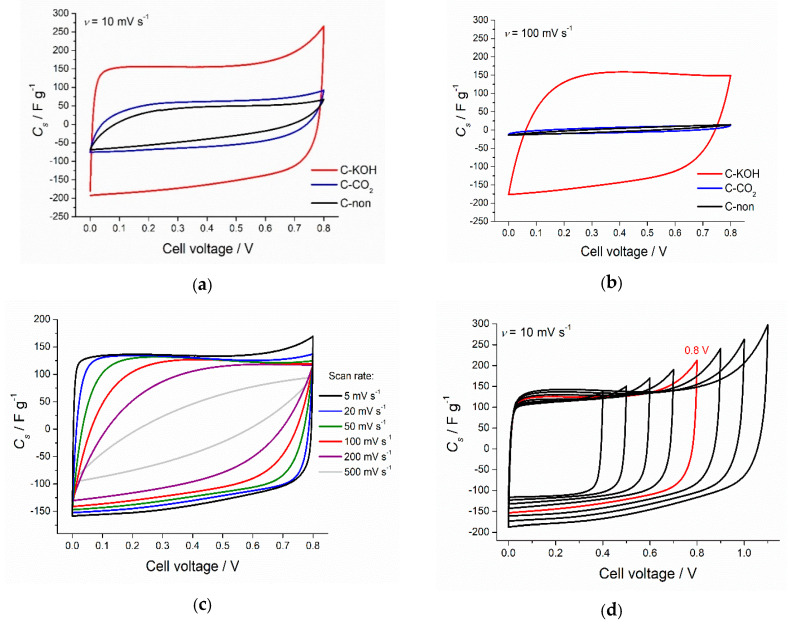
CV curves of the chestnut-derived activated carbons recorded at 10 mV s^−1^ (**a**), and at 100 mV s^−1^ (**b**). CV curve for chemically activated carbon (3:1 by weight with KOH) recorded at different scan rates (**c**), and for various potential windows (**d**), CV at 0.8 V (marked red) was selected for further experiments in the two-electrode setup.

Figure 3a,b show the CV curves recorded for biochar without activation (sample marked as C-non), biochar after physical activation (C–CO_2_) and KOH-activated biochar (C–KOH). Activation process increases the capacitance values of the investigated carbon samples. The increase is more pronounced in the case of C–KOH sample, which is related to the exceptionally high surface area. Chemical activation of carbon materials leads to the formation of micropores, and it has been well demonstrated that the ion confinement in the micropores can lead to the higher capacitance [50,51,52,53]. The increase of the scan rate from 10 mV s^−1^ to 100 mV s^−1^ leads to the significant distortion of the CV curve shape and the essential decrease of capacitance values for C-non and C–CO_2_ samples. At the same time, CV curve of C–KOH preserves almost unchanged rectangular shape at 100 mV s^−1^, which indicates good capacitive behavior of KOH-activated carbon. This result proves that more developed porosity causes better penetration of the electrolyte through the sample.

Cyclic voltammograms recorded for C–KOH at various scan rate are presented in Figure 3c. CV shape up to 20 mV s^−1^ is ideally rectangular, at 50–100 mV s^−1^, the sample still exhibits excellent capacitive behavior. In contrast, at scan rates higher than 200 mV s^−1^, the diffusion limitation is noticed, resulting in the deterioration of the ideal rectangular shape. Elemental composition of our AC samples revealed relatively high oxygen content (see Table 1), indicating the presence of oxygen-rich surface functional groups, which can contribute to the capacitance values. However, the CV curve at low scan rate exhibits a rectangular shape with no visible redox activity. Pseudocapacitive behavior coming from oxygen-rich functional groups such as quinone/hydroquinone would show broad redox peak in CV curve of a symmetric capacitor at about 0.35 V in 6 M KOH as presented by Fic et al. [54]. Moreover, the oxygen content was calculated as a difference to 100%, assuming no other elements present. In the case of C–KOH sample, some potassium impurities might have remained in the micropores, which may have caused an overestimation of oxygen content. Based on electrochemical data, we assume that the contribution from the surface functional groups is not so significant and the essential part of the capacitance value comes from the electric double layer formation.

Based on the CV curves of C–KOH at 10 mV s^−1^ for different cell voltages (Figure 3d), 0.5 V and 0.8 V were selected for further electrochemical tests. 0–0.8 V voltage range was the maximum, where our symmetric capacitor operated without a drastic capacitance decrease upon cycling.

**Figure 4:**
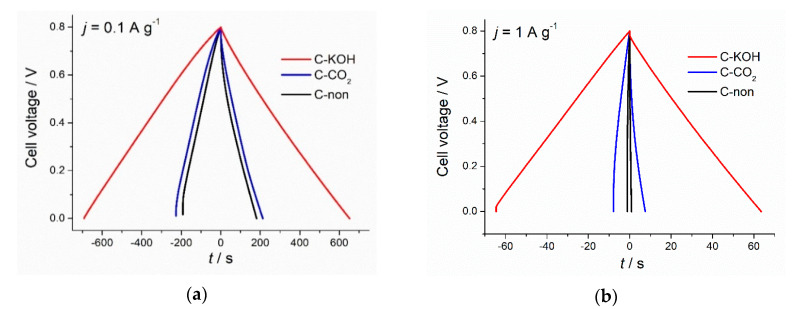
Charge-discharge profiles of C–KOH, C–CO_2_ and C-non samples at 0.1 A g^−1^ (**a**) and 1 A g^−1^ (**b**).

**Figure 5:**
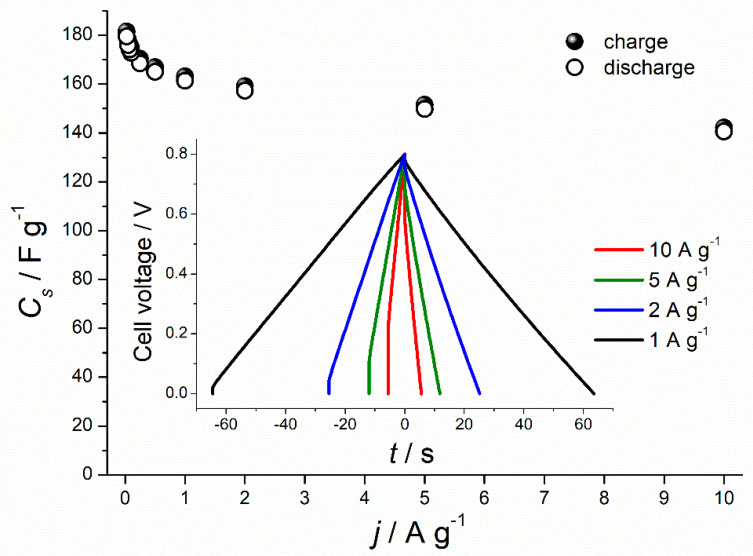
Specific capacitance values recorded for C–KOH sample at different current densities. Inset: galvanostatic charge-discharge (GCD) profiles at high current densities (1–10 A g^−1^).

As shown in Figure 4a, galvanostatic charge-discharge profiles of all three types of carbons, recorded at a low current density of 0.1 A g^−1^, had triangular symmetrical distribution. The lowest specific capacitance of 57.4 F g^−1^ was recorded for non-activated carbon and slightly higher capacitance of 67.30 F g^−1^ for CO_2_-activated carbon (increase by 17%). Chemical activation of the chestnut-derived carbon with KOH, in turn, led to the 3-fold increase of the discharge capacitance (173 F g^−1^) in comparison to non-activated carbon and a 2.5-fold increase compared to the physically activated carbon. However, at higher current density (1 A g^−1^) (Figure 4b) only C–KOH sample preserves the triangular shape of GCD curve, proving that it has good capacitive properties for electrochemical capacitors in contrast to C–CO_2_ and C-non samples. The specific capacitance values of non-activated, CO_2_- and KOH-activated carbons, calculated from the GCD upon polarization with 1 A g^−1^, are equal to 2.8 F g^−1^, 24.5 F g^−1^ and 161 F g^−1^, respectively. C–KOH exhibits the highest specific capacitance compared to C–CO_2_ and C-non, which is mainly attributed to the well-developed porosity of this sample and the resulting high specific surface area. C–KOH sample, contrary to C-non and C–CO_2_, shows outstanding rate capability (see Figure 5 and Figure S1 in Supplementary Materials). Its specific capacitance hardly decreases with an increase of the current density, and at 10 A g^−1^ reaches 140 F g^−1^. Moreover, the IR drop (in GCD curve recorded at 1 A g^−1^) for C–KOH is significantly smaller (30 mV) compared to C–CO_2_ (245 mV) and C-non (350 mV) samples. IR drop refers to the potential induced by the resistance of the electrode/electrolyte interface. Lower IR, achieved for the KOH-activated chestnut-derived carbon, results from the microporosity introduced to the material, which creates diffusion paths for the ions, hence, lowers the resistance at the interface between electrode and electrolyte.

**Figure 6:**
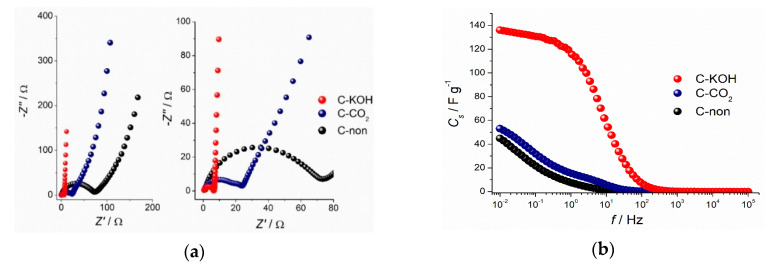
Nyquist plots (**a**) and dependence of the specific capacitance from the frequency (**b**) of the investigated carbon samples.

The Nyquist plots, representing imaginary part (−*Z*”) versus real part (*Z*’) of the impedance recorded at the open-circuit voltage for C–KOH, C–CO_2_ and C-non samples, are given in Figure 6a. A semicircle at a high frequency region, representing charge transfer resistance (*R*_CT_), is present for all the samples. The *R*_CT_ value is the highest for the non-activated carbon and decreases for the activated samples characterized by the higher porosity, resulting in the enhanced diffusion of the electrolyte. The activation process improves the capacitive character of the samples, which is observed as a change of the curve slope in the low-frequency region for activated samples—the more vertical the slope, the better capacitive behavior of the electrode. In case of non-activated carbon, a line at an angle of 45° is seen, which is related to the diffusion-controlled processes. Interestingly, no such feature can be found for C–KOH sample, which presents a vertical line, indicating the purely capacitive response of the electrode material. Well-developed porosity of the KOH-activated carbon material provides channels for the electrolyte; hence diffusion of ions is not a limiting process. The Nyquist plot shape of C–CO_2_ sample reveals an intermediate character between C-non and C–KOH samples, which is consistent with the electrochemical results obtained by CV and GCD techniques.

Based on the EIS measurements, the specific capacitance *C*_s_ of the investigated electrode materials was evaluated using the Equation (3):

(3) $$C_{s}=2\cdot \frac{1}{2\pi \cdot f\cdot \left(-Z”)\right)\cdot m}$$

where: *f* is the frequency (Hz), *−Z*” is the imaginary part of the impedance (Ω) and *m* is the active mass of one electrode (g).

Capacitance values obtained from the impedance spectroscopy are consistent with those calculated from cyclic voltammetry and galvanostatic charge-discharge measurements. Specific capacitance values drop at high frequencies due to too short time for ions to access the whole carbon surface. In other words, part of the surface of the activated carbon, especially deep in the pores is not reached by the ions, and therefore is ionically more resistive. The capacitance dependence on the frequency presented in Figure 6b proves that the porosity of the C–KOH sample is far more developed than of the C–CO_2_ and C-non samples.

**Figure 7:**
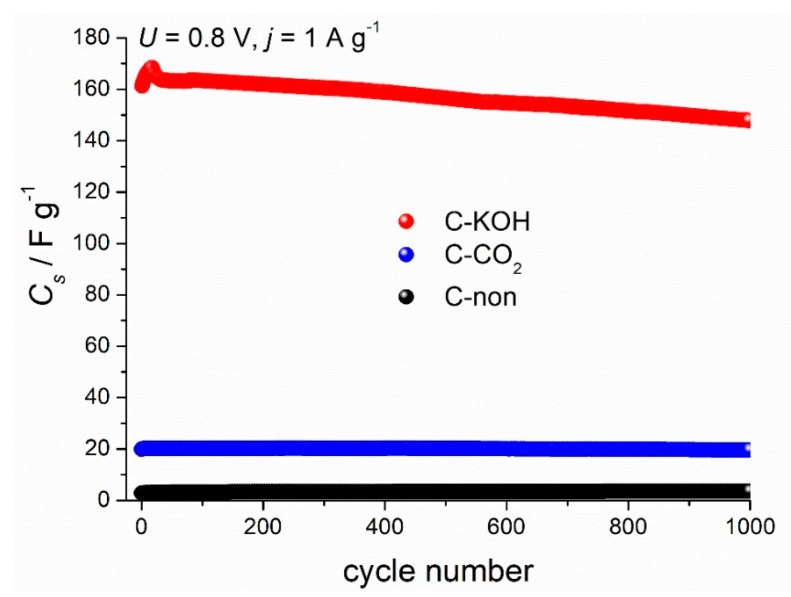
Electrochemical performance of C–KOH activated carbons; 6 M KOH electrolyte; GCD measured at 1 A g^−1^ and 0.8 V cell voltage.

Another essential aspect for the supercapacitor performance is the cycling stability that determines the lifetime of the device. Capacitance values of three symmetric capacitors prepared from the carbon samples (C–KOH, C–CO_2_ and C-non) during 1000 galvanostatic charge-discharge cycles are shown in Figure 7. The capacitance of the C–KOH electrode decreases after 1000 cycles by 7.8%.

**Figure 8:**
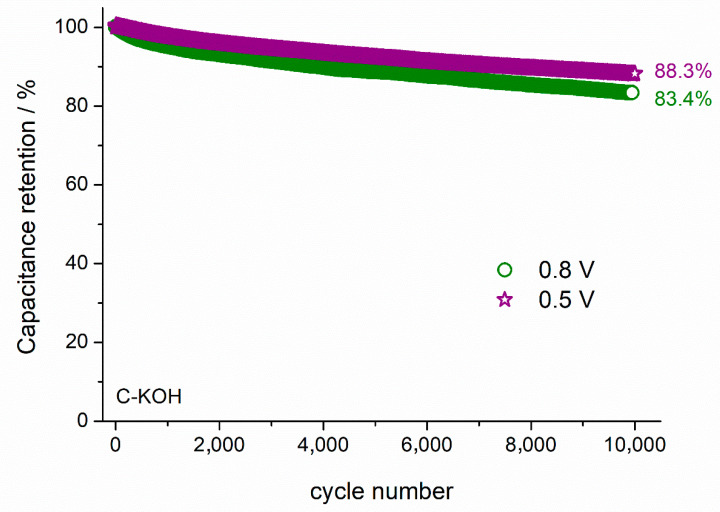
The capacitance retention dependence on the cell voltage during 10,000 GCD cycles at 1 A g^−1^ recorded for the symmetric capacitor with C–KOH electrodes.

The capacitance retention of the capacitor can be improved by reducing the cell voltage. As presented in Figure 8, the capacitance retention after 10,000 cycles is equal to 83.4 and 88.3%, when the cell operates at 0.8 and 0.5 V, respectively. Specific energy (*E*) and specific power (*P*) of the symmetric electrochemical capacitor were calculated from the Equations (4) and (5), respectively:

(4) E= 12CU2,

where: *C*—capacitance of the device (calculated per active mass of both electrodes) and *U*—cell voltage.

(5) P= EΔt,

where Δ*t*—time of the discharge from galvanostatic charge/discharge.

**Figure 9:**
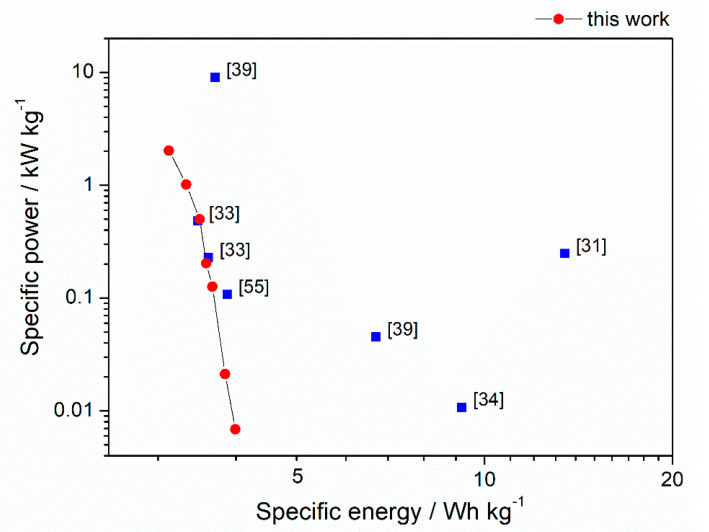
Ragone plot showing the comparison between the specific energy and power obtained for activated carbon from horse chestnut seeds (this work) and chestnut-shells (literature).

The highest capacitance recorded at 0.1 A g^−1^ for C–KOH sample was equal to 173 F g^−1^. The value is moderate compared to other biomass-derived activated carbons reported in the literature (see Table 3). However, as mentioned above, our C–KOH sample is characterized by outstanding rate capability, which is crucial for the high-power application. The energy density values achieved for our chestnut seed-derived AC are average compared to AC derived from chestnut [31,33,34,39,55], as shown in Ragone plot (Figure 9). However, the energy and power densities should be calculated for the system, which is close to a real device as they are essential parameters from the technological point of view. Our electrodes were in the form of pellets with mass loading of approximately 10 mg cm^−2^, which is high compared to other works [31,33,34,35,55,56]. In many papers, the electrodes are deposited on nickel foam with very low mass loading of 1.5–5 mg cm^−2^ [31,33,34,35,55,56], which improves charge propagation and diffusion of the electrolyte through the electrode. Besides, Ni foam as a current collector in the strong alkaline medium may contribute to the capacitance values due to the oxidation of nickel to Ni(OH)_2_ and NiOOH [57,58,59]. Thus, some reported values are considerably higher than one could expect for the same material working in the real system. Moreover, there are mistakes in literature reports regarding calculation of capacitance of the cell. The authors sometimes recalculate the values per mass of single electrode instead of mass of both electrodes, which make the reported *C*, *E* and *P* values significantly overestimated (e.g., [35,56]).

Furthermore, preparation of biomass-derived activated carbons varies significantly in literature reports and often involves complicated and expensive multistage procedures. It is worth noticing that biomass type is important for the morphology, structure, porosity and surface area of the resulted char. Thermal treatment and activation conditions are other parameters that are crucial for the characteristics of the final carbon material. Although a lot of work has been done so far on biomass-derived activated carbons, it is still important to explore this topic in order to convert biomass into a useful product.

## 4. Conclusions

Here we demonstrate, for the first time, the use of horse chestnut seeds-derived activated carbons as electrode material for electrochemical capacitors. Chestnut-derived biochar was activated by two methods, the physical one by CO_2_ treatment, and the chemical one using KOH. BET and SEM analyses confirmed high surface area and well-developed porosity of the KOH activated sample. Electrochemical measurements using cyclic voltammetry, galvanostatic charge-discharge and impedance spectroscopy gave comparable results. The high capacitance value of 160 F g^−1^ (at 1 A g^−1^) outstanding rate capability of 140 F g^−1^ at 10 A g^−1^ and good cycling stability (83.4% of initial capacitance after 10,000 GCD cycles) were achieved. The specific energy of 3.12 Wh kg^−1^ at a specific power of 2030 W kg^−1^, make the obtained AC a promising candidate as electrode material for supercapacitors, which is one of the potential applications of biomass-derived activated carbons of well-developed surface area.

## Figures and Tables

**Table 1:** Elemental analysis of the raw chestnut, biochar after pyrolysis and activated carbons.

Sample	Elemental Analysis (wt.%)				
	C	H	N	S	O *
Chestnut, raw material	45.97	6.65	2.53	0.2	44.65
Chestnut, biochar (C-non)	75.14	1.32	2.41	0.0	21.13
CO_2_ activated carbon (C–CO_2_)	75.04	1.11	2.61	0.0	21.24
KOH activated carbon (C–KOH)	60.54	0.85	2.63	0.0	35.98

* Calculated as the difference to 100% assuming no other elements present.

**Table 2:** The Brunauer-Emmett-Teller (BET) surface area (S_BET_) and total pore volume (V_p_) of the activated carbon obtained from the chestnut.

Sample	C:Activating Agent Ratio	S_BET_ (m^2^ g^−1^)	V_p_ (cm^3^ g^−1^)
C-non	-	17.1	0.0094
C–CO_2_	1:0.5 *	105.7	0.056
C–KOH	1:3 **	1221.2	0.625

* molar ratio, ** mass ratio.

**Table 3:** Comparison of specific surface area (SSA) and capacitance values for activated carbons derived from various biomass types, treated with different pyrolysis and activation conditions.

Biomass Type	Thermal Treatment	Activation Process (C: Activated Agent Ratio, Temp., Time)	SSA/m^2^ g^−1^	C/F g^−1^ (in 6 M KOH, Symmetric Device)	Reference
Tobacco rods	HTC * (200 °C, 12 h, autoclave)	1:3 C:KOH, 800 °C, 1 h	2115	263 @ 0.5 A g^−1^	[60]
Cornstalk core	Pre-carbonization 300 °C 2 h; pyrolysis 800 °C 3 h	1:6 C:KOH, 800 °C, 3 h	2139	186.8 @ 2 A g^−1^	[61]
Rice bran	Pyrolysis 700 °C	1:4 C:KOH, 850 °C, 1 h	2475	323 @ 0.1 A g^−1^	[62]
Ginkgo shells	Pyrolysis 600 °C	1:2 C:KOH, 700 °C, 1 h; 1% Co(NO_3_)_2_ for 12 h; 900 °C for 2 h	1775	237 @ 2 mV s^−1^	[32]
Celtuce	Pyrolysis 600 °C	1:4 C:KOH, 800 °C, 1 h	3404	273 @ 0.5 A g^−1^	[63]
Broad beans	Carbonization 800 °C	1:3 C:KOH, 650 °C, 1 h	655	202 @ 0.5 A g^−1^ measured in 3-electrode cell	[64]
Pistachio shell	Pyrolysis 750 °C	1:3 C:KOH, 750 °C, -	1069	261 @ 0.2 A g^−1^	[65]
Rice husk	Carbonization 450 °C, 1 h	1:4 C:KOH, 800 °C, several hours	3145	367 @ 2.27 A g^−1^	[66]
Rice husk	1:1.6 (C: NaOH) 400 °C, 4 h	1:5 C:KOH, 850 °C, 1 h	2696	147 @ 0.1 A g^−1^	[67]
Lignin	1000 °C, 15 min (1 °C min^−1^ to 400 °C and 2 °C to 1000 °C)	1:2 C:KOH, 1000 °C, ramp rate 10 °C min^−1^	1148	91.7 @ 2 mV s^−1^, measured in 3-electrode cell	[68]
Rotten carrot	100 °C, 24 h	1:2 C:ZnCl_2_, 900 °C, 2 h	1155	137 @ 10 mV s^−1^	[69]
Starch	Pretreatment with 10 wt.% (NH_4_)_2_HPO_4_ aqueous solution, 210 °C, 3 h; carbonization 600 °C, 2 h	1:4 C:KOH, 800 °C, 2 h	3251	304 @ 0.05 A g^−1^	[70]
Chestnut shell	Drying 80 °C	1:2 C:ZnCl_2_, 700 °C, 1.5 h	1987	105.4 @ 0.1 A g^−1^	[33]
Chestnut shell	Freeze-dried, 12 h	1:0.25 C:melamine, 800 °C, 2 h	691.8	402.8 @ 0.5 A g^−1^ measured in 3-electrode cell	[35]
Chestnut shell	90 °C, 24 h	1.8:1 C:K_2_SO_4_, 800 ° C, 2 h	1412	265 @ 0.1 A g^−1^ measured in 3-electrode cell	[34]
Chestnut shell	60 °C, 2 h	1:3 (C:KHCO_3_), 850 °C;2.5, 2 h	2298	387 @ 2A g^−1^ measured in 3-electrode cell	[31]
Chestnut shell	1 M HNO_3_, 24 h	1:2.5, 750 °C, 4 h	1347.9	174 @ 0.5 A g^−1^	[55]
Horse chestnut seed	Pyrolysis 800 °C, 30 min	1:3, 800 °C, 1 h	1252.5	173 @ 0.1 A g^−1^	This work
				161 @ 1 A g^−1^	
				140 @ 10 A g^−1^	

* HTC—hydrothermal carbonization.

## Supplementary Materials

The following are available online at https://www.mdpi.com/1996-1944/13/20/4658/s1. Figure S1. Specific capacitance values recorded for C-non (**a**) and C–CO_2_ (**b**) samples at different current densities. Inset: GCD profiles at high current densities (1–10 A g^−1^).
