# Supplementary material for: Chestnut-Derived Activated Carbon as a Prospective Material for Energy Storage
Source: Materials (Basel). 2020 Oct 19;13(20):4658. doi: 10.3390/ma13204658 (PMC7603389; doi:10.3390/ma13204658)
Supplement: Supplementary file 1 [file materials-13-04658-s001.pdf]

materials

MDPI

Supplementary materials

Chestnut-Derived Activated Carbons as a Perspective Material for Energy Storage

Katarzyna Januszewicz [1], Anita Cymann-Sachajdak [1], Paweł Kazimierski [2], Marek Klein [2], Justyna Łuczak [3] and Monika Wilamowska-Zawłocka [1,*]

[1] Department of Energy Conversion and Storage, Faculty of Chemistry, Gdańsk University of Technology, Narutowicza 11/12, 80-233 Gdańsk, Poland; katjanus@pg.edu.pl (K.J.); anita.cymann@pg.edu.pl (A.C.-S.)
[2] Institute of Fluid Flow Machinery, Polish Academy of Sciences, 80-233 Gdańsk, Poland; pkazimierski@imp.gda.pl (P.K.); marek.klein@imp.gda.pl (M.K.)
[3] Department of Process Engineering and Chemical Technology, Faculty of Chemistry, Gdańsk University of Technology, Narutowicza 11/12, 80-233 Gdańsk, Poland; justyna.luczak@pg.edu.pl
* Correspondence: monika.wilamowska@pg.edu.pl; Tel.: +48-58-347-24-74

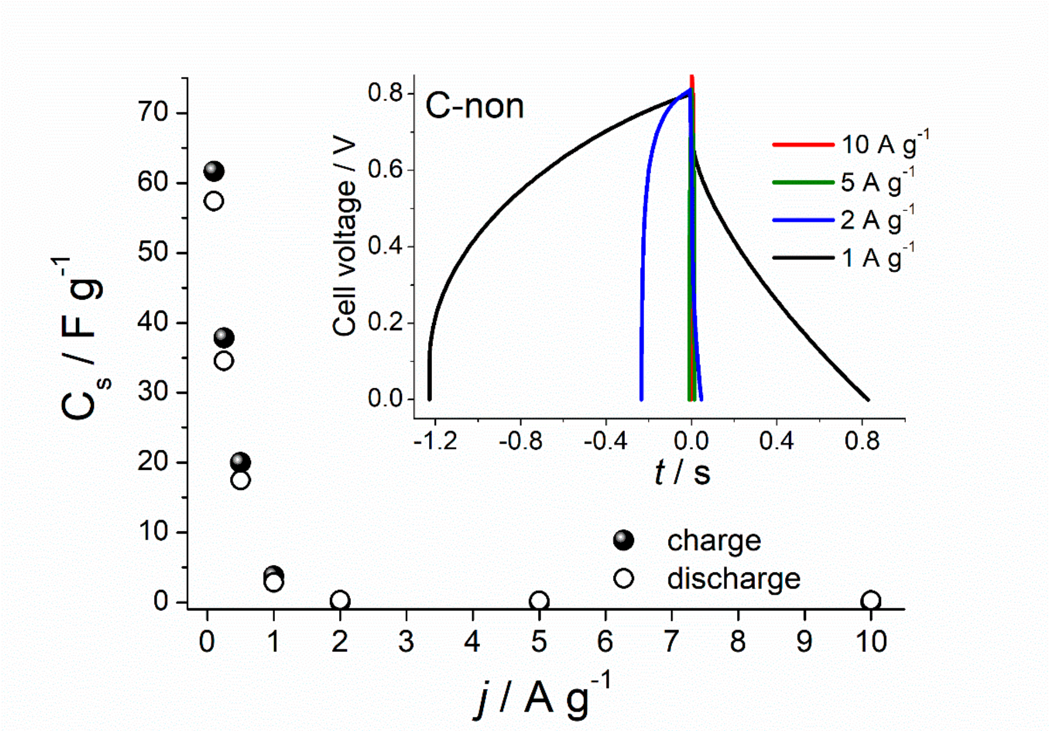

(a)

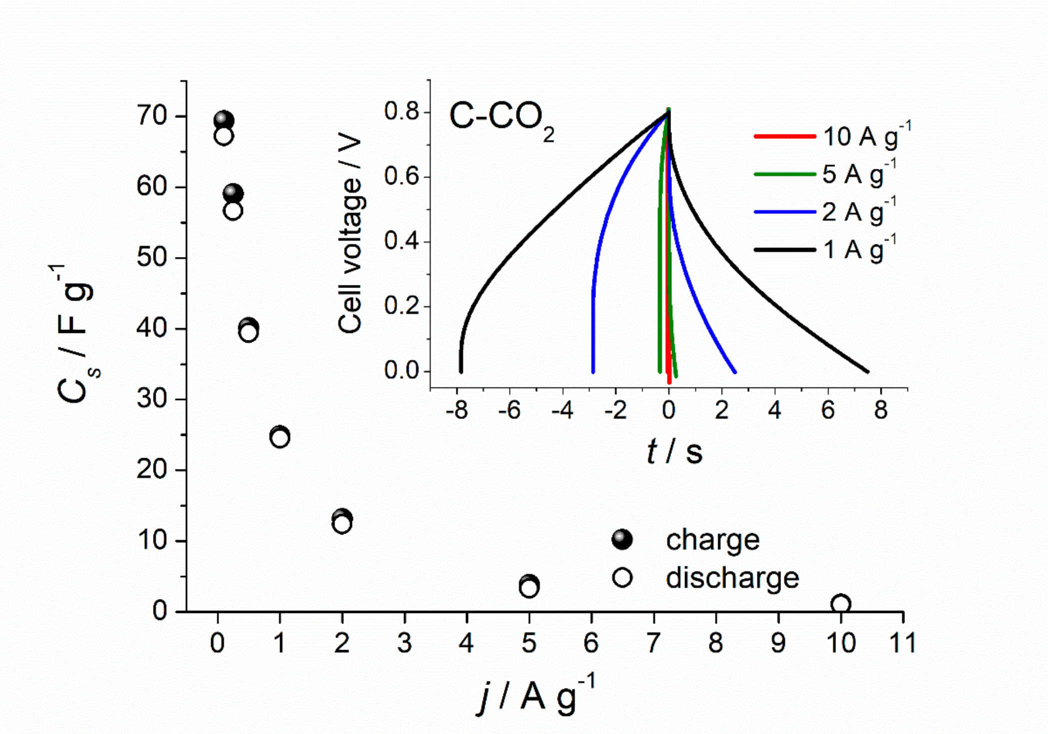

(b)

13 **Figure S1**. Specific capacitance values recorded for C-non (**a**) and C-CO_2 (**b**) samples at different
14 current densities. Inset: GCD profiles at high current densities (1-10 A g^{-1}).
